# Sleep apnea prevalence and severity after coronary revascularization versus no intervention: a systematic review & meta-analysis

**DOI:** 10.1007/s11325-024-03164-4

**Published:** 2024-11-27

**Authors:** Marjo Ajosenpää, Satu Sarin, Tero Vahlberg, Ulla Ahlmen-Laiho, Peker Yüksel, Nea Kalleinen, Jenni Toivonen

**Affiliations:** 1 Department of Pulmonary Diseases and Clinical Allergology, Sleep Research Center, University of Turku, Turku, Finland; 2 Department of Anesthesiology and Intensive Care, University of Turku, Turku, Finland; 3 Division of Perioperative Services, Intensive Care and Pain Medicine, Turku University Hospital, Turku, Finland; 4 Department of Biostatistics, University of Turku and Turku University Hospital, Turku, Finland; 5 Heart Center, Turku University Hospital, University of Turku, Turku, Finland; 6 Department of Pulmonary Medicine, Koc University School of Medicine, Istanbul, TR 34010 Turkey; 7 Department of Molecular and Clinical Medicine, Sahlgrenska Academy, University of Gothenburg, Gothenburg, SE 40530 Sweden; 8 Department of Clinical Sciences, Respiratory Medicine and Allergology, Faculty of Medicine, Lund University, Lund, SE 22185 Sweden; 9 Division of Pulmonary, Allergy, and Critical Care Medicine, University of Pittsburgh School of Medicine, Pittsburgh, PA 15213 USA

**Keywords:** CAD, coronary artery disease, OSA, obstructive sleep apnea, PCI, CABG

## Abstract

**Purpose:**

Obstructive sleep apnea (OSA) is a common disease in patients with coronary artery disease (CAD). Approximately 40–80% of cardiovascular disease patients have obstructive sleep apnea. The manifestation of it can vary significantly in different types of CAD patients. This systematic review and meta-analysis investigate the prevalence and severity of OSA in patients with acute coronary syndrome (ACS).

**Methods:**

This systematic review was conducted according to PRISMA guidelines. The first inclusion criteria were that a reliable sleep study had to be done after treating the patients’ acute coronary incident. All patients in the studies included were adults suffering from an ACS who underwent either coronary artery bypass grafting surgery (CABG), a percutaneous coronary intervention (PCI) or had no invasive coronary intervention done. A search was conducted within four valid databases 27.1.2023 and all suitable articles published after 1.1.2010 were included.

**Results:**

Eight studies fulfilled the full inclusion criteria. In five of them, a sleep study had been performed after PCI, in two after no coronary intervention, and in one study after CABG. Mean AHI in no-OSA group after PCI was 9.5 /h (95% CI 5.3–13.7) and in the no intervention group 6.4 /h (95% CI 3.5–9.4). In OSA patients, mean AHI after PCI was 34.9 /h (95% CI 25.9–43.8) vs. 24.1 /h without intervention (95% CI 15.6–32.6).

**Conclusions:**

Sleep apnea is very common among ACS patients and should be screened for and addressed after the acute coronary intervention. Moreover, we found that OSA is more severe in patients in whom PCI for ACS was indicated as opposed to patients who underwent no coronary intervention.

**Supplementary Information:**

The online version contains supplementary material available at 10.1007/s11325-024-03164-4.

## Introduction

Untreated obstructive sleep apnea (OSA) is known to cause various pathophysiological changes in the human body and increase both morbidity and mortality [1–3]. The worldwide prevalence of OSA is alarmingly high, and it is estimated that at least 936 million men and women between the ages 30 and 69 have mild to severe OSA (apnea-hypopnea index, AHI; measured as events/hour, ≥ 5) [4]. The prevalence of sleep apnea has increased over time [5]. Up to 17% of men and 9% of women with 50–70 years of age suffer from moderate to severe sleep apnea (AHI ≥ 15/h), but are either unaware or aware of their diagnosis [5]. This subgroup of the OSA population (AHI ≥ 15/h) is clinically important as they are the ones for which treatment would be recommended even in the absence of associated symptoms or secondary disorders [6]. Symptomatic sleep apnea with AHI ≥ 5/h and excessive daytime sleepiness is referred to as OSA syndrome and its prevalence has been estimated to be 6% of all men and 4% of all women [7].

Even though OSA has been shown to be an independent risk factor for the development of coronary artery disease (CAD), especially in middle-aged individuals [8, 9], studies have indicated that up to 37–64% of patients who undergo coronary artery bypass grafting (CABG) suffer unknowingly from at least of a moderate form of OSA [10–14]. Acute coronary syndrome (ACS) patients undergoing the less invasive percutaneous coronary intervention (PCI) also suffer from OSA with an estimated prevalence of 35–62% [15]. The rate of underdiagnosis in these CAD patients with OSA is concerning because several studies have linked OSA to significant postoperative complications after revascularization [16–24] However, it has not been investigated whether the revascularization technique or the procedure itself affects the severity or prevalence of OSA.

The aim of this systematic review is to examine what prior research has established about OSA in ACS patients, as well as determine the differences between the prevalence and severity of OSA in different intervention groups (those undergoing PCI or CABG and those without coronary intervention).

## Methods

This systematic review and meta-analysis followed the accepted guidelines for reporting of systematic reviews and meta-analysis (PRISMA). Ethical approval was not required as the systematic review was based on secondary data. The review protocol was registered in the PROSPERO database (registration number: CRD42023418669).

### Search strategy

The search was conducted within four different reliable databases (Pubmed, Google Scholar, ScienceDirect and Cochrane) on January 27, 2023. The search strategy, including the terms used, was formulated with a committed research librarian as well as a colleague experienced in systematic meta-analysis. The period set for the timing of publication was between 1.1.2010 and 1.12.2023. The records were limited to include the form of randomized controlled trials. The search terms used were “coronary artery disease”, “percutaneous coronary intervention”, “coronary artery bypass grafting surgery”, “obstructive sleep apnea” and different versions of these terms (Appendix A). Additional searches were performed on September 7th, 2023, and on May 26th, 2024 and they verified that no new articles on the topic had been published. All the results were uploaded onto the Zotero platform. The screening of all titles, abstracts, and full texts was performed by two reviewers (MA and SS) and this screening process was documented utilizing the Google Forms Sheets platform.

### Inclusion and exclusion criteria

The inclusion and exclusion criteria were defined before the searches were conducted. The Inclusion criteria were as follows: (1) adult patients with ACS who underwent either a CABG, a PCI or no invasive coronary intervention, (2) the revascularization procedure was performed because of the CAD, (3) a reliable sleep investigation was performed on the patients (for example polysomnography (PSG) or reliable sleep monitoring at home) after the revascularization.

**Fig. 1:**
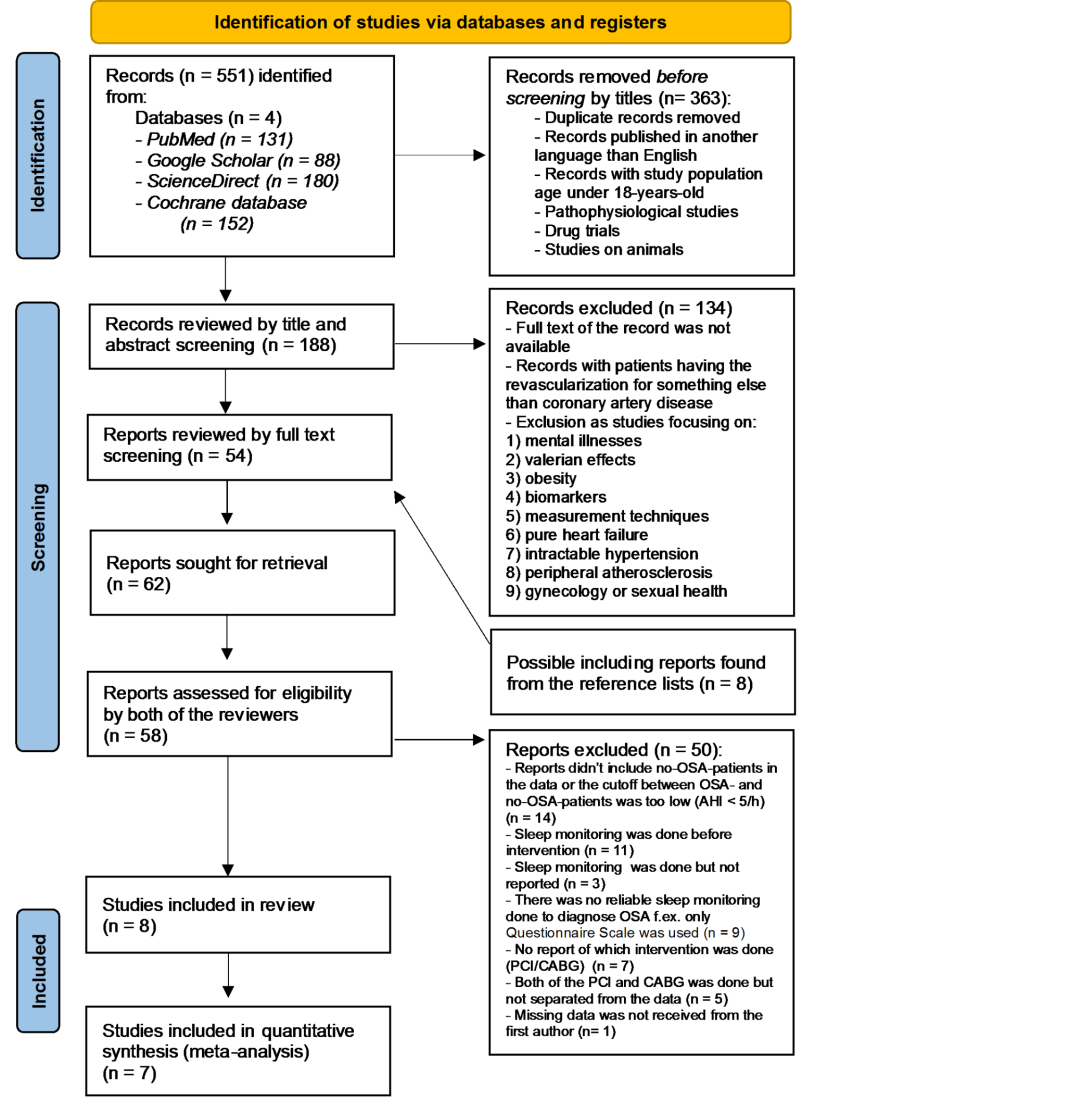
PRISMA flow diagram. OSA, obstructive sleep apnea; AHI, apnea-hypopnea index; PCI, percutaneous coronary intervention; CABG, coronary artery bypass grafting surgery. Meta-analysis, Lowest oxygen saturation level in obstructive sleep apnea

Exclusion criteria were: (1) the patient population in the study was under the age of 18, (2) the study was published in a language other than English, (3) the indication for a revascularization intervention was peripheral arteriosclerosis and not CAD, (4) pathophysiological studies, (5) drug trials, and (6) animal studies. For a detailed list of all exclusion criteria see Fig. 1 and Appendix B.

### Screening

In the first step of study selection, all duplicates were removed. Then, articles where it was obvious from the title alone that an exclusion criterium was met were excluded. The studies remaining after these steps were distributed equally to two reviewers (MA and SS) for independent screening. Any potential ambiguities regarding the inclusion were reviewed and discussed with the second author, and disagreements were presented to a third reviewer (JT). Once an undisputed selection of included studies was established, both reviewers screened all the full texts of those articles to survey whether additional exclusion criteria were met.

At this stage, records were excluded if a reliable sleep study was performed but not reported or it was not stated whether it was done before or after intervention. A study was also excluded if it could not be established which intervention was performed (this missing data was requested from the first author and if no reply was received, the study was rejected), both PCI and CABG were performed. A detailed list of excluded articles can be found in Appendix B.

In three selected studies, the sleep monitoring was performed as polysomnography in a sleep laboratory, in three other studies with portable device at home, and two of the studies in hospital with portable device (Table 1). The subjects were divided into OSA or no-OSA groups based on their AHI scores by the sleep studies. American Academy of Sleep Medicine (AASM) has published the International Classification of Sleep Disorders (3rd Edition) where the cutoffs for mild, moderate and severe OSA presents AHI being 5.0-14.9 /h; AHI 15.0-29.9 /h, and AHI ≥ 30 /h [25]. Most of the included studies classified OSA-patients as having an AHI ≥ 15 /h and no-OSA as AHI < 15 /h. Exceptions included a study conducted by Lee [16] where the cutoff in AHI was ≥ 30 /h and a study by Schiza [26] AHI ≥ 10 /h.

**Table 1:** Study characteristics

First author (year)	Study design	Country	Sample size *n*	PCI/ CABG/No	Outcome	Age (years) mean (SD)	Male sex *n* (%)	BMI (kg/m^2^) mean (SD)	Sleep monitoring (device)	PSG done after ACS (days)	AHI cutoff (events /h)	OSA vs. no-OSA *n* (%)	AHI OSA vs. no-OSA (events /h) mean (SD) *p* value	SpO_2_ lowest OSA vs. no-OSA (%) mean (SD) *p* value
Buchner et al. (2022)	POS *(sub-analysis)*	Germany	41	PCI	Patho-physiology of the heart	*56 (10) **54 (11)	35 (85.4)	*29.3 (3.4) **26.7 (2.5)	PSG (Alice System)	4 ± 1	≥ 15	21 (51.2) 20 (48.8)	31 (13) 6 (3) *p* = 0.001	83 (4) 86 (4) *p* = 0.100
Calcaianu et al. (2019)	POS	France	53	PCI	Patho-physiology of the heart	59 (9.6)	43 (81.1)	28.5 (4.2)	PSG (Cidelec)	60	≥ 15	39 (73.6) 14 (26.4)	42.3 (13.4) 15.1 (3.9) *p* < 0.001	- - -
Lee et al. (2011)	PLCS	Singapore	105	PCI	MACE	52.7 (9.8)	103 (98)	24.9 (3.3)	Home (Somte)	3.5 ± 1.5	≥ 30	44 (42) 61 (58)	48.6 (13.4) 14.4 (8.2) *p* < 0.001	- - -
Liu et al. (2022)	POS	China	119	PCI	Patho-physiology of the heart	*55.4 (12.0) **56.3 (11.2)	104 (87.4)	*26.9 (3.4) **25.4 (2.9)	Hospital, portable device (ApneaLink Air)	(during hosp- italiza- tion)	≥ 15	60 (50.4) 59 (49.6)	28.7 (5.25) 7.5 (1.33) *p* < 0.001	82.4 (6.9) 86.6 (4.6) *p* = 0.016
Loo et al. (2014)	POS	Asian (multi-center)	68	PCI	MACCE	54.2 (8.8)	59 (86.8)	25.5 (3.8)	Home (Embletta)	14	≥ 15	24 (35.3) 44 (64.7)	24.0 (8.78) 4.9 (3.65) *p* < 0.001	82.5 (7.75) 89.0 (5.75) *p* < 0.001
Peker et al. (2022)	RCT *(sub analysis*)	Sweden	147	CABG	Post-operative atrial fibrillation	*65.3 (7.8) **63.5 (9.5)	107 (86.3)	*28.5 (3.9) **25.4 (3.6)	Home (Embletta)	73 ± 30	≥ 15 < 5	106 (72.1) 18 (12.2)	32.3 (16.3) 3.2 (1.3)	79.3 (9.1) 86.5 (8.8)
Schiza et al. (2012)	LS	Greece	52	No	Prevalence and time course of OSA	55.8 (13)	40 (76.9)	28.5 (4.9)	PSG (Alice 5)	3	≥ 10	28 (53.8) #22 (42.3)	19.7 (6.9) #4.9 (1.93)	84.5 (3.7) -
Zhang et al. (2023)	POS	China	590	No	MACCE	*59.0 (9.8) **56.8 (10.0)	470 (79.7)	*28.07 (3.58) **26.12 (3.51)	Hospital, portable device (ApneaLink Air)	(during hospi- taliza- tion)	≥ 15	288 (48.8) 302 (51.2)	28.4 (14.07) 7.9 (4.44) *p* < 0.001	82 (6.67) 87 (2.96) *p* < 0.001

POS, prospective observational study; PLCS, prospective, longitudinal cohort study; RCT, randomized controlled trial; LS, longitudinal study; PCI, percutaneous coronary intervention; CABG, coronary artery bypass grafting surgery; No, no coronary intervention; MACE, major adverse cardiac event; MACCE, major adverse cardiac and/or cerebrovascular event; BMI, body mass index; PSG, polysomnography; ACS, acute coronary syndrome; AHI, apnea-hypopnea index; AHI cutoff, participants divided in OSA- or no-OSA-groups based on sleep study results; OSA, participants who suffer from obstructive sleep apnea based on sleep study results; no-OSA, participants who do not have obstructive sleep apnea based on sleep study results; SpO2 lowest, the lowest score of oxygen saturation based on sleep study results

Age and BMI reported only in *OSA- and **no-OSA-groups, not together [29–31, 38]. #Schiza did not report no-OSA-group’s sleep study results but they were in their different report [32]

### Data extraction

All data was stored and sorted into an Microsoft Office Excel file, where the extraction and final inclusion were performed. Two independent researchers (MA and SS) conducted initial data collection on the outcomes of each article. These records were listed using a standardized chart to extract data and assess study quality. Extracted information included publication title, first author, publication year, study design, study population, age and gender distribution, PCI/CABG/no (coronary) intervention, PSG findings (AHI and lowest oxygen saturation level (SpO_2_) value during night), observation time, outcomes and possible confounding factors, as well as factors reducing the quality of the research. Any missing relevant data was requested from the first author of the studies. Additional data was received from M.D. PhD Y. Peker of the CABG population.

### Quality assessment

The quality of the published studies was evaluated on the principles and procedural guidelines of systematic reviews presented by Egger [27]. The Newcastle-Ottawa scale was used to assess the methodological quality of the selected studies. Both reviewers screened independently all included records using this 8-criteria checklist classifying the studies from low (7–9 points) to moderate (4–6 p.) or high risk (0–3 p.) of bias. The strength of the epidemiological evidence for the data in each record was rated as high when the *p* value was < 0.001. The evidence was rated moderate when the *p* value was < 0.05 but > 0.001, and weak with *p* values above 0.05. The reference lists of all included studies were manually evaluated, and the reliability was evaluated individually.

### Statistical analysis

The data from all included records was separately evaluated. The study characteristics for continuous variables, which followed normal distribution, were summarized with mean and standard deviation (SD), whereas non-normally distributed variables as median and interquartile range (IQR) or range. Categorical variables were summarized with counts (n) and percentages. The standard deviations (SD) and standard errors (SE) were calculated with an established method from the range or the interquartile range when they were not directly reported in the articles with the supposition that the data follows normal distribution [28].

The heterogeneity of the studies was estimated by the I^2^-test, where I^2^ > 50% was considered to signify high heterogeneity. The Cochran’s Q test was used to examine statistical heterogeneity between subgroups (significant at *p* < 0.05). Assessment of publication bias was performed with visual evaluation of the funnel plots. A sensitivity analysis was conducted using a leave-one-out approach. All the statistical analyses were performed using IBM SPSS Statistics (version 29.0.2.0, IBM Corp., Armonk, NY). Only reported data was analyzed and no data was imputed except from the timing of performing the sleep monitoring after revascularization. Studies that reported sleep monitoring timing to be “during the hospital stay” were assumed to be done approximately on the 7th day after the revascularization [29, 30].

## Results

### Search results

The flow chart of study selection is shown in Fig. 1. Altogether, 551 articles were identified in the initial searches, out of which a total of 8 articles were included in the systematic review and 7 in the meta-analysis.

### Study characteristics

All in all, the selected studies contained a pool of 1 175 subjects with a weighted age average of 57.7 years (SD 2.8) and a weighted BMI average of 26.8 kg/m^2^ (SD 0.75). The total number of males was 961 (81.8%). Because their sleep monitoring findings had not been reported, twenty-five subjects were excluded from the review. In the report of CABG participants (*n* = 23) with AHI between 5 and 15 /h were excluded from meta- analysis, because there were OSA-patients with graded AHI being ≥ 15 /h and no-OSA with AHI < 5 /h [31]. Two participants (*n* = 2) were excluded from the no-OSA group in the study by Schiza [32]. In the end, a total of 1 150 subjects were included in the final analysis.

The characteristics of the included studies are presented in Table 1. Sleep monitoring was performed after PCI in 5 studies (with a total of 386 participants), after no coronary intervention in two (642 participants) and after CABG for unstable CAD in only one (147 participants) [31]. ACS was defined as ST-segment elevation myocardial infarction (STEMI), non-STEMI (NSTEMI) or unstable angina pectoris (UAP) following the current standard clinical guidelines [33]. Studies where there was no coronary intervention were either designed to compare the prevalence of OSA after revascularization intervention and non-revascularization [30] or after acute myocardial infarction before revascularization [26, 32].

### OSA after ACS

The selected studies contained a total of 610 (53.0%) participants who had OSA (OSA group) and 540 (47.0%) participants without OSA (no-OSA group). The mean AHI in the no-OSA group after PCI was 9.5 /h (95% CI 5.3–13.7) and in the no intervention group 6.4 /h (95% CI 3.5–9.4). The pooled effect size for AHI from all of the OSA groups was 31.7 /h (95% CI 24.2–39.3) and for the no-OSA groups 7.9 /h (95% CI 4.9–10.9). OSA prevalence after PCI was 48.7% and the OSA patients’ mean AHI was 34.9 /h (95% CI 25.9–43.8) in median 7 days (range 3.5–60). In the no intervention group 49.2% had OSA, and their mean AHI was 24.1 /h (95% CI 15.6–32.6) in median 5 days (range 3–7). In the only study where the sleep investigation had been performed after CABG, 83% of the patient pool had OSA and the mean AHI of OSA patients was 32.3 /h after an average of 73 days.

In the group of patients with sleep apnea, bypass surgery patients were older (mean age 65.3 years; SD 7.8) and had a higher BMI (mean 28.5 kg/m^2^; SD 3.9) than the patients who either underwent a PCI or had no coronary intervention. In the PCI group, the mean age was 55.4 years (SD 1.8) and mean BMI 26.6 kg/m^2^ (95% CI 25.0- 28.2). The mean age of the study subjects in the no intervention group mean was 59 (SD 0.3) and mean BMI 27.4 kg/m^2^ (95% CI 23.2–31.6).

The lowest SpO_2_ value was not included in the Lee [16], Schiza [26, 32] and Calcaianu [34] studies, but where that lowest value in the OSA group was reported, it was 82.3% (95% CI 80.5–84.1) and 87.0% (95% CI 85.6–88.5%) in the non-OSA group. The variation of registered sleep registration timing after ACS was high (from a few days to 3 months). The mean lowest saturation level (SpO_2_) in the OSA group after PCI was 82.7% (95% CI 81.8–83.4). In group no coronary intervention OSA was median 83.2% (95% CI 80.7–85.6%) and after CABG was 79.3%. Table 2 compares sleep monitoring results in the three patient groups.

**Table 2:** Sleep monitoring results in different intervention groups

Intervention (PCI/CABG/no)	PCI	CABG	No coronary intervention
Sample size, *n*	386	147	642
OSA group			
*n* (%)	188 (48.7)	106 (72.1)	316 (49.2)
age (years)	55.2 ± 1.8	65.3 ± 7.8	59.0 ± 0.3
BMI (kg/m^2^)	26.6 ± 3.6	28.5 ± 3.9	27.4 ± 4.2
AHI (events /h)	34.9 ± 4.6	32.3 ± 16.3	24.1 (19.7-28.4)
lowest SpO_2_ (%)	82.5 (82.4-83.0)	79.3 ± 9.1	83.3 (82.0-84.5)
no-OSA group			
*n* (%)	198 (51.3)	18 (12.2)	324 (50.4)
age (years)	54.0 ± 9.9	63.5 ± 9.5	57.4 ± 11.0
BMI (kg/m^2^)	25.5 ± 3.1	25.4 ± 3.6	26.6 ± 3.8
AHI (events /h)	9.6 ± 4.8	3.2 ± 1.3	6.4 (4.9-7.9)
lowest SpO_2_ (%)	86.6 (86.0-89.0)	86.5 ± 8.8	87.0*

Results presented with categorical variables as counts (n) and percentages, continuous variables as mean (SD) and non-normally distributed variables as median (interquartile range; IQR)

PCI, percutaneous coronary intervention; CABG, coronary artery bypass grafting surgery; OSA, participants who suffer from obstructive sleep apnea based on the sleep study result; no-OSA, participants who do not have obstructive sleep apnea based on sleep study results; AHI, apnea-hypopnea index; lowest SpO_2_, the lowest score of oxygen saturation based on sleep study results

### Meta-analysis

Appendix C contains the funnel plots, and appendix D contains a summary of the Newcastle-Ottawa risk of bias scoring for each article. Based on the funnel plots, publication bias was significant in both the PCI and the no coronary intervention groups. The main reason for this appeared to be the difference of the AHI cutoff between the no-OSA and OSA subgroups. High heterogeneity between studies likely arises from the differing timing of the sleep study, varying from a median of 7 days (range 3.5 to 60 days) after PCI to a median of 5 days (range 3 to 7 days) after no coronary intervention.

Sufficient data for meta-analysis was reported for the lowest SpO_2_ and AHI levels in OSA patients after PCI and in the no intervention subgroup. CABG patients were not included in the meta-analysis because there only one such study was found. The effect sizes for SpO_2_ were 84.9 (SD 0.6) for the after PCI group and 84.5 (SD 0.0) for the no intervention group.

### Meta-analysis, AHI in OSA patients

**Fig. 2:**
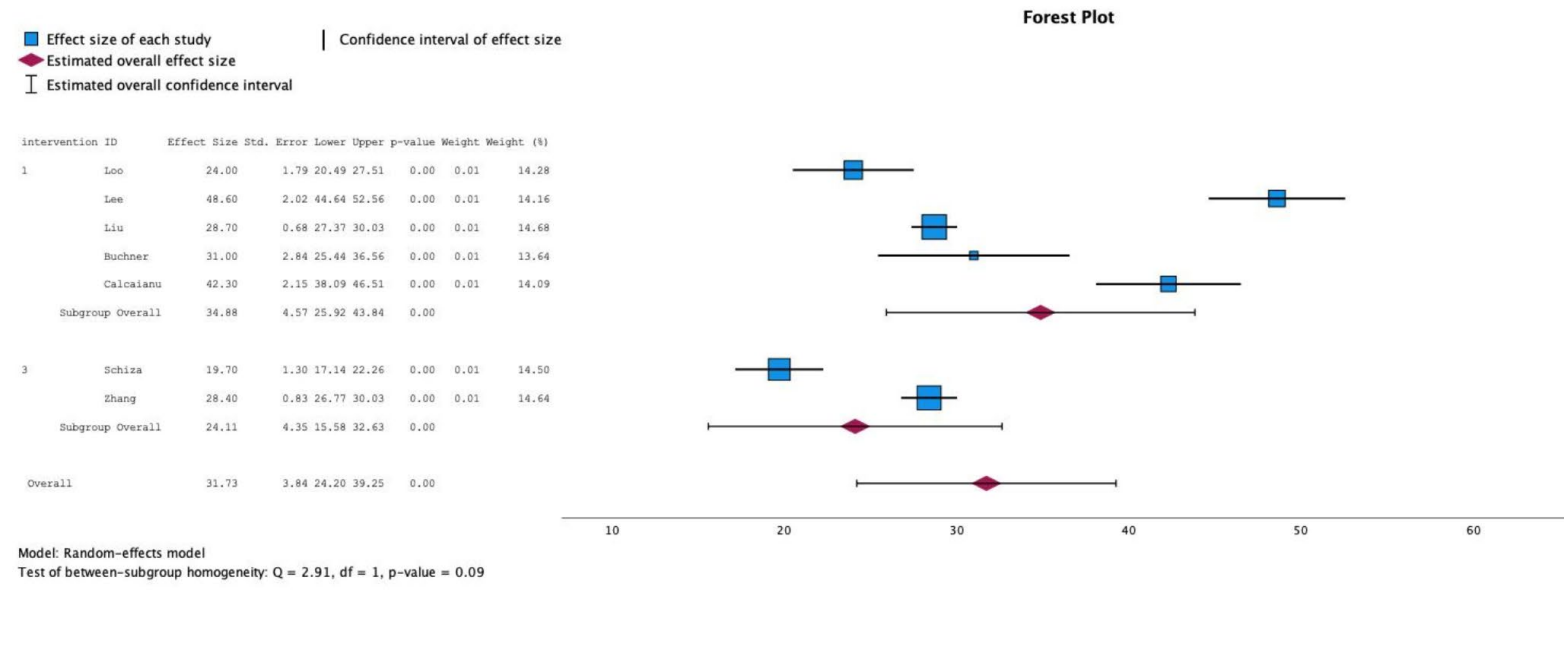
Forest plot comparing obstructive sleep apnea patients’ apnea-hypopnea-index (AHI, events/h) between percutaneous coronary intervention (PCI; intervention 1) and no intervention (intervention 3) groups. AHI in PCI group was more severe (mean 34,9 /h) than in no intervention group (mean 24.1 /h). Meta-analysis, apnea-hypopnea-index (AHI) in obstructive sleep apnea

Heterogeneity was tested with a Q-test, which was statistically insignificant (*p* = 0.09) in the no intervention group unlike in the group that underwent PCI. Meta-analysis of OSA patients revealed that, after ACS, the AHI was lower in patients with no intervention than in patients who underwent PCI: 24.1/h (95% CI 15.6–32.6; I^2^ = 98.6%) vs. 34.9 /h (95% CI 25.9–43.8; I^2^ = 97.4%), (Fig. 2).

### Meta-analysis, lowest SpO2 in OSA patients

**Fig. 3:**
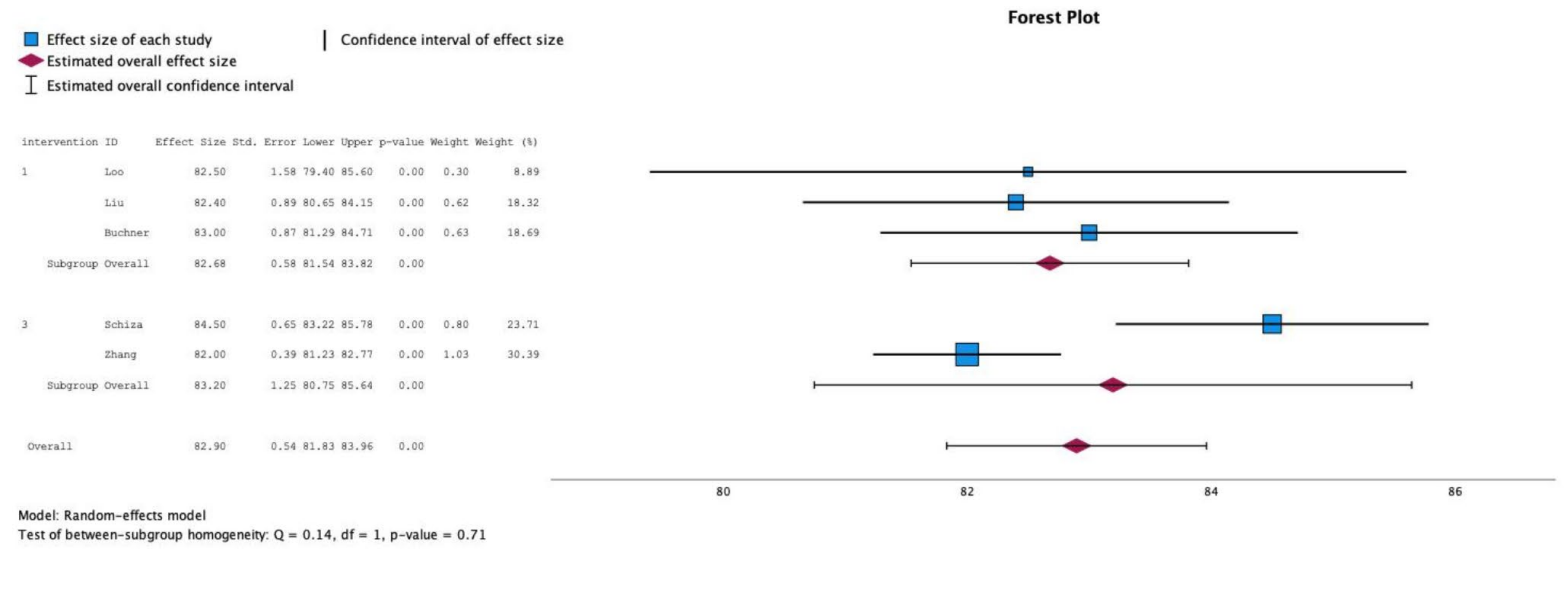
Forest plot comparing obstructive sleep apnea patients’ lowest oxygen saturation level (SpO_2_, %) during study night between percutaneous coronary intervention (PCI; intervention 1) and no intervention (intervention 3) groups. Lowest saturation during nighttime in PCI group (mean 82,7%) is lower than in no intervention group (mean 83,2%). Meta-analysis, Lowest oxygen saturation level in obstructive sleep apnea

Among OSA patients, only five studies reported the lowest SpO_2_ from a performed sleep study. In OSA patients who underwent PCI, the lowest SpO_2_ was 82.6% (95% CI 81.8–83.4%; I^2^ = 0) vs. 83.2% (95% CI 80.8–85.6; I^2^ = 90.7%) in OSA patients with no coronary intervention. Heterogeneity between these subgroups was not significant (*p* = 0.71). (Fig. 3).

## Discussion

This systematic review and meta-analysis shows that approximately half of ACS patients have OSA, and the severity of OSA varies between different patient groups. OSA patients who had no coronary intervention as a treatment for their ACS had lower AHI. The lowest saturation level in PCI group was higher than in the no intervention group, although pertinent data was available only for three studies, and the significance was not statistically relevant. Both groups are, however, suffering from low saturation levels during nighttime.

The combination of repetitive apneas, arousals from sleep, and intermittent hypoxia are referred to as sleep disordered breathing (SDB), of which OSA is a subtype, and these patients are particularly vulnerable after acute myocardial infarction. The increased cardiac workload and endothelial dysfunction may ultimately result in a mismatch of SDB patients’ oxygen supply and oxygen demand after revascularization. A study done by Arzt et al. [35] showed that, despite successful revascularization, patients with acute myocardial infarction who have sleep-disordered breathing had predisposing factors for heart failure such as a bigger likelihood of prolonged myocardial ischemia than those without sleep-disordered breathing [35].

Sleep apnea can be of either obstructive or central type. In OSA the airway is obstructed, while in central sleep apnea (CSA), there is an arrest of breathing with no respiratory efforts caused by a halt at the central respiratory drive [36]. Due to the different pathophysiological mechanism, these sleep apnea types possibly differ in terms of the complications they cause after ACS. Previous research done by Tafelmeier et al. shows that observed CSA before elective CABG can cause more major pulmonary complications, such as respiratory failure or pneumonia after surgery [37].CSA increased also the length of ICU or intermediate care stay, and prolonged stay at hospital [37]. In this review, most of the included studies used CSA as an exclusion criterion for study participants. However, one study that also reported central AHI, showed that obstructive but not central respiratory events, were associated with diastolic dysfunction after myocardial infarction [38].

OSA patients are more likely are more likely to be treated with CABG than PCI [39]. However, when AHI ≥ 30 /h and the OSA is classified as severe, OSA participants are more likely to be treated with CABG [40]. Patients with OSA had more comorbidities, an increased risk of postoperative pneumonia and their hospital stays were longer than those of coronary patients without OSA [39]. CABG is usually done in the acute phase of unstable CAD and PSG is therefore difficult to schedule before the intervention. One study showed that the easy and fast STOP-BANG questionnaire could predict pulmonary complications after CABG, identifying up to 36.1% of at-risk patients [41]. The STOP-BANG questionnaire has five-point questions on snoring, tiredness, observations by others of breathing problems during nighttime, high blood pressure and whether the subject’s BMI is over 35 kg/m^2^.

In the randomized, controlled RICCADSA-trial [31], the prevalence of OSA was higher than that previously reported, and majority of the participants did not report daytime sleepiness. Worryingly, two-thirds of these patients had OSA, a higher incidence than other known risk indicators in this cohort for CAD such as hypertension, diabetes, or current smoking [11]. This reveals the importance of screening for OSA after revascularization — it should be considered as a part of secondary prevention.

When comparing these three groups — especially the PCI and no coronary intervention groups — it is impossible to say unequivocally whether OSA patients who undergo revascularization are more multimorbid than patients who receive no invasive intervention and thus have more severe apnea. It can be assumed, however, that their disease pathophysiology differs in some way from the non-revascularization group. Patients with severe CAD are the likeliest to be recommended revascularization and PCI is the modality of choice for myocardial reperfusion [42]. CABG is usually offered to hospitalized patients with ACS and a higher comorbidity burden [43]. In the Zhang [30] study, revascularization was not considered in patients without significant coronary artery stenosis (≤ 70% diameter stenosis) but they highlighted that this did not mean an absence of lesions in coronary arteries. Their data showed that, in the non-revascularization group, OSA patients had significantly higher incidence of atherosclerotic coronary vessels, and OSA was associated with higher risk of subsequent cardiovascular events in patients without revascularization. In this review, OSA patients who had a PCI were younger and had lower BMI values than those who did not undergo revascularization, but there was no significant difference between these characteristics. However, such a finding suggests that the higher incidence of OSA in these patients is not only related to traditional OSA risk factors (BMI and age), but also to the severity and type of CAD. Further investigations on whether OSA patients have more apneic episodes during the night after revascularization are needed.

The difficulty in diagnosing CAD patients with OSA stems from most of suffering from non-sleepy type apnea and thus many of them have not been diagnosed [44]. This is worrisome because when OSA patients’ AHI is over 30, this elevates the risk of cardiovascular disease and their all-cause mortality is 46% higher than for healthy individuals [45]. A meta-analysis done by Yu [46] showed that even when patients are using a continuous positive airway pressure (PAP) machine, the occurrence of MACCE’s (major adverse cardiac and/or cerebrovascular event) did not decrease later in life in OSA patients. Although there are other benefits of treatment with a PAP machine for sleep apnea, Yu et al.‘s findings did not support the notion that death or the prevention of cardiovascular outcomes should be adopted as an indication for such treatment [46].

This review and meta-analysis demonstrates a large degree of heterogeneity in currently published studies with varying criteria of timing to perform sleep monitoring and diagnosing OSA in ACS patients. In the studies meeting the inclusion criteria, all except one explored OSA less than two weeks after ACS. Schiza [26] showed in their study that there is a high prevalence of OSA in the acute phase of ACS, but this did not persist, and AHI was significantly lower six months later, indicating that OSA may be transient. They suspected that a stunned myocardium and progressing heart failure after ACS could be causing tissue swelling in the upper airways, worsening the obstruction. A need exists for further well-designed prospective studies with a long-term follow-up after ACS to fully answer this question.

Many studies have shown how OSA affects the progress of cardiovascular disease, quality of life and mortality. Still, there are many open questions, such as the causal relationships between OSA and CAD and the effect of coronary interventions on the prognosis of OSA patients in later life. To our knowledge, there have been no previous systematic reviews investigating the prevalence and severity of OSA after ACS and potential differences between patients in these intervention groups. More research is needed to reveal how ACS and its treatment is affecting patients’ sleep disorders and apnea tendency later in life.

### Strengths and limitations

The strength of our study is the accurate screening: we only included articles that reported the results of reliable sleep monitoring after ACS. Many articles were excluded because sleep monitoring was performed before ACS. A significant limitation in our study was the small number of reliable studies found, and the fact that only one study of the CABG patients fulfilled the inclusion criteria. The main challenge of this meta-analysis arose from the significant heterogeneity between studies and how the timing of sleep monitoring was not always reported clearly. We tested removing one significantly longer follow-up [34] study from the meta-analysis, and the results did not differ from our original analysis.

## Conclusion

As many as 53% of ACS patients in the included studies had OSA, and the mean AHI in all OSA groups was 31.9 /h, indicating severe sleep apnea. We thus conclude that the possibility of OSA should be evaluated as a part of a CAD patient’s treatment process. The severity of OSA appears to vary in different patient groups, and a particularly significant number of patients who have undergone PCI or CABG suffer from severe OSA. More studies are needed to determine how the two different types of sleep apnea, central or obstructive, affect comorbidities and complications after ACS.

## Electronic supplementary material

Below is the link to the electronic supplementary material.

Supplementary Material 1

Supplementary Material 2

Supplementary Material 3

Supplementary Material 4

Supplementary Material 5

## Data Availability

Supplementary data to this article can be found online at Google drive file.

## Declarations

This result on this systematic review and meta-analysis indicates that OSA should be actively screened for in patients with ACS and identified OSA risk factors. Follow-up with polysomnogaphy studies after the acute phase of the coronary syndrome could also prove beneficial.
