# Correction to: Sleep apnea prevalence and severity after coronary revascularization versus no intervention: a systematic review & meta-analysis

**DOI:** 10.1007/s11325-025-03273-8

**Published:** 2025-02-18

**Authors:** Marjo Ajosenpää, Satu Sarin, Tero Vahlberg, Ulla Ahlmen-Laiho, Peker Yüksel, Nea Kalleinen, Jenni Toivonen

**Affiliations:** 1 Department of Pulmonary Diseases and Clinical Allergology, Sleep Research Center, University of Turku, Turku, Finland; 2 Department of Anesthesiology and Intensive Care, University of Turku, Turku, Finland; 3 Division of Perioperative Services, Intensive Care and Pain Medicine, Turku University Hospital, Turku, Finland; 4 Department of Biostatistics, University of Turku and Turku University Hospital, Turku, Finland; 5 Heart Center, Turku University Hospital, University of Turku, Turku, Finland; 6 Department of Pulmonary Medicine, Koc University School of Medicine, Istanbul, TR 34010 Turkey; 7 Department of Molecular and Clinical Medicine, Sahlgrenska Academy, University of Gothenburg, Gothenburg, SE 40530 Sweden; 8 Department of Clinical Sciences, Respiratory Medicine and Allergology, Faculty of Medicine, Lund University, Lund, SE 22185 Sweden; 9 Division of Pulmonary, Allergy, and Critical Care Medicine, University of Pittsburgh School of Medicine, Pittsburgh, PA 15213 USA

**Sleep and Breathing (2024) 29:13**

10.1007/s11325-024-03164-4

In the original version of this article, the given and family names of authors were incorrectly structured as: Ajosenpää Marjo, Sarin Satu, Vahlberg Tero, Ahlmen-Laiho Ulla, Yüksel Peker, Kalleinen Nea, Toivonen Jenni.

The correct names of authors are: Marjo Ajosenpää, Satu Sarin, Tero Vahlberg, Ulla Ahlmen-Laiho, Peker Yüksel, Nea Kalleinen, Jenni Toivonen.

The original article has been corrected.

