# Supplementary material for: Sleep apnea prevalence and severity after coronary revascularization versus no intervention: a systematic review & meta-analysis
Source: Sleep Breath. 2024 Nov 27;29(1):13. doi: 10.1007/s11325-024-03164-4 (PMC11602854; doi:10.1007/s11325-024-03164-4)
Supplement: Supplementary file 1 — Supplementary Material 1 [file 11325_2024_3164_MOESM1_ESM.docx]

Appendix A. The search terms

Table A. 1. The search terms used in each database

| Database | Search terms |  |
| --- | --- | --- |
| PubMed | "coronary artery disease [Mesh]" AND ("coronar* arter* bypass" OR "Coronary Artery Bypass"[Mesh] OR "heart bypass" OR "cabg") OR (" Percutaneous Coronary Intervention" [Mesh] "percutaneous" AND "coronar*" OR "heart" AND "intervent*" OR "PCI") AND ("respirator* disorder*" OR "sleep apn*" OR "obstructive sleep apn*" OR "sleep apnea, obstructive" [Mesh] OR "Sleep Apnea Syndromes"[Mesh] OR "Respiration Disorders"[Mesh]) |  |
| Google Scholar | "coronary artery disease" AND ("respirator* disorder*" OR "sleep apn*" OR "obstructive sleep apn*" OR "sleep apnea, obstructive" OR "Sleep Apnea Syndromes") AND ("coronar* arter* bypass" OR "Coronary Artery Bypass" OR "cabg" OR " Percutaneous Coronary Intervention" OR "percutaneous coronar*" OR "PCI") AND ("clinical trial" OR "randomized controlled trial") |  |
| ScienceDirect | "coronary artery disease" AND ("obstructive sleep apn?a" OR "Sleep Apnea Syndromes" OR "Sleep disord! breath!") AND ("coronar! arter! bypass" OR "cabg" OR "percutaneous coronar! intervention" OR "PCI") |  |
| Cochrane database | (coronar* NEXT arter* NEXT bypass OR heart NEXT bypass OR cabg OR PCI OR percutaneous NEXT coronar* NEXT intervent* OR coronar* NEXT revascular*) AND (sleep NEXT apne* NEXT syndrom* OR sleep NEXT apne* OR respirati* NEXT disord*) |  |
