# Supplementary material for: Sleep apnea prevalence and severity after coronary revascularization versus no intervention: a systematic review & meta-analysis
Source: Sleep Breath. 2024 Nov 27;29(1):13. doi: 10.1007/s11325-024-03164-4 (PMC11602854; doi:10.1007/s11325-024-03164-4)
Supplement: Supplementary file 2 — Supplementary Material 2 [file 11325_2024_3164_MOESM2_ESM.docx]

Appendix B. More detailed additional exclusion criteria

Table B. 1. List of additional exclusion criteria

|  | Additional exclusion criteria |
| --- | --- |
| 1 | Treatment interventions, for example pre-operational evaluation |
| 2 | Heart failure |
| 3 | Hypertension |
| 4 | Pathophysiological research |
| 5 | Obesity and sleep disorders |
| 6 | Peripheral atherosclerosis |
| 7 | Gynecologic patients |
| 8 | Children |
| 9 | Pre-clinical studies and medicine research |
| 10 | Device research |
| 11 | Transplant patients |
| 12 | Valve surgery and OSA |
| 13 | Anatomical disorders, for example pectus excavatum |
