# Supplementary material for: Sleep apnea prevalence and severity after coronary revascularization versus no intervention: a systematic review & meta-analysis
Source: Sleep Breath. 2024 Nov 27;29(1):13. doi: 10.1007/s11325-024-03164-4 (PMC11602854; doi:10.1007/s11325-024-03164-4)
Supplement: Supplementary file 3 — Supplementary Material 3 [file 11325_2024_3164_MOESM3_ESM.docx]

Appendix D. Risk of bias assessment


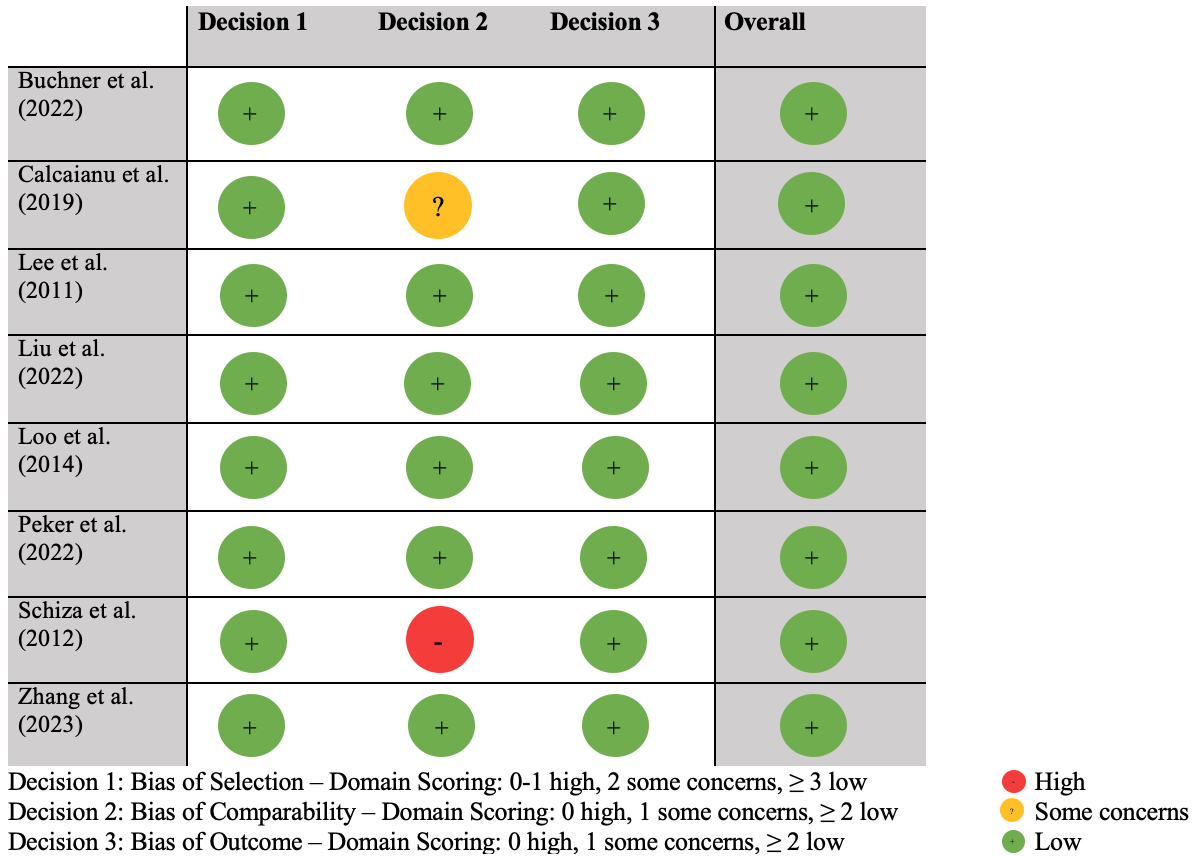


Figure D. 1. Risk of bias assessment of each article using Newcastle-Ottaw
