# Supplementary material for: Sleep apnea prevalence and severity after coronary revascularization versus no intervention: a systematic review & meta-analysis
Source: Sleep Breath. 2024 Nov 27;29(1):13. doi: 10.1007/s11325-024-03164-4 (PMC11602854; doi:10.1007/s11325-024-03164-4)
Supplement: Supplementary file 4 — Supplementary Material 4 [file 11325_2024_3164_MOESM4_ESM.docx]

 Table B. 2. List of excluded articles that might appear to meet the inclusion criteria

|  | **Excluded article/study**  First author (publication year) | **Reason for exclusion** |
| --- | --- | --- |
| 1 | Ooi (2023) | The participants had angina but did not have obstructive Coronary Artery Disease based on the coronary angiography |
| 2 | Uchôa (2015) | The sleep study (polysomnography and portable monitoring) was performed before CABG |
| 3 | Low (2013) | The study population included stable CAD participants who did not have a revascularization procedure |
| 4 | Summerer (2021) | The study population was divided based to CRS Grading of Coronary Collaterals and the OSA or no-OSA participants was not separated from the data |
| 5 | Sánchez-de-al-Torres (2020) | There was no statement which revascularization technique was done to the participants |
| 6 | Nakashima (2013) | The AHI cutoff between the OSA and no-OSA participants was ≥ 5 events/h |
| 7 | Berger (2013) | Participants were stratified according to oxygen desaturation index (ODI) into two subgroups and there was no data of AHI results |
| 8 | Glantz (2012) | There was both PCI and CABG done in the study population but there was no division between these groups based on revascularization technique |
| 9 | Morra (2017) | There was PCI, CABG and medical intervention done in the study population but there was no division between these groups based on revascularization technique |
| 10 | Lee (2010) | The sleep study results were not reported (AHI or lowest SpO2) |
| 11 | Fan (2019) | There was both PCI and CABG done in the study population but there was no division between these groups based on revascularization technique |
| 12 | Bauça (2017) | There was no statement which revascularization technique was done to the participants |
| 13 | Cheong (2021) | There was no statement which revascularization technique was done to the participants |

CABG, coronary artery bypass grafting surgery; CAD, coronary artery disease; CRS, Cohen‐Rentrop Score; OSA, participants who suffer from obstructive sleep apnea based on the sleep study result; no-OSA, participants who do not have obstructive sleep apnea based on sleep study results; AHI, apnea-hypopnea index; AHI cutoff, participants divided in OSA- or no-OSA-groups based on sleep study results; PCI,  percutaneous coronary intervention; lowest SpO2, the lowest score of oxygen saturation based on sleep study results; SDB, sleep-disordered
