# Supplementary material for: Sleep apnea prevalence and severity after coronary revascularization versus no intervention: a systematic review & meta-analysis
Source: Sleep Breath. 2024 Nov 27;29(1):13. doi: 10.1007/s11325-024-03164-4 (PMC11602854; doi:10.1007/s11325-024-03164-4)
Supplement: Supplementary file 5 — Supplementary Material 5 [file 11325_2024_3164_MOESM5_ESM.docx]

Appendix C. Funnel plots


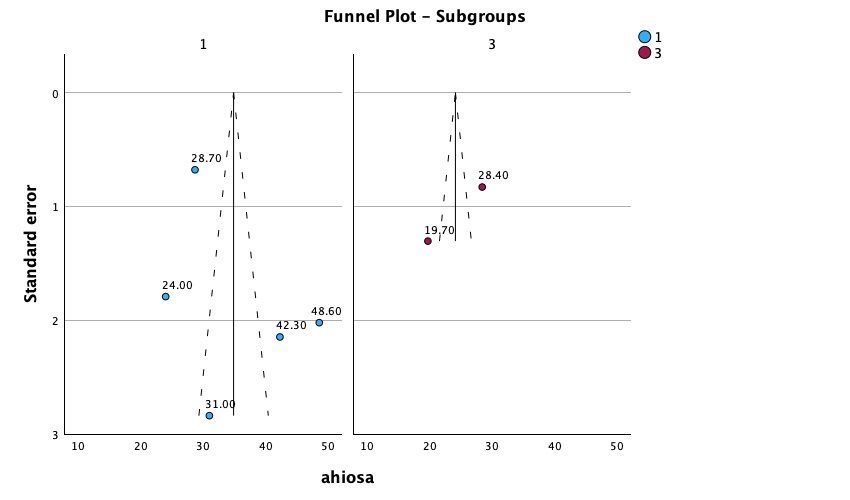


Figure C. 1. Funnel plot 1, apnea-hypopnea-index (AHI) in obstructive sleep apnea patients describing heterogeneity according to the distribution of standard errors of individual studies

AHI, apnea-hypopnea index; AHIOSA, participants who suffer from obstructive sleep apnea based on the PSG result and their AHI; PSG, polysomnography

Subgroups 1 = PCI, percutaneous coronary intervention; 3 = No intervention


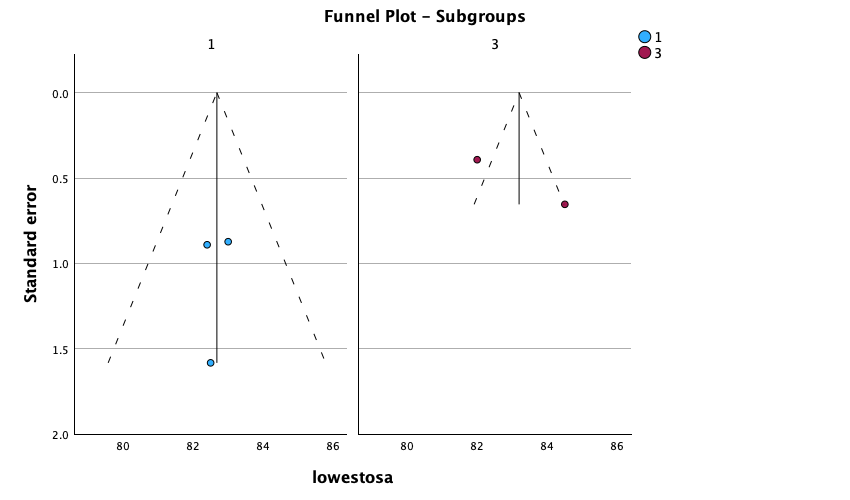


Figure C. 2. Funnel plot 2, Lowest oxygen saturation level in obstructive sleep apnea patients describing heterogeneity according to the distribution of standard errors of individual studies

Lowestosa, participants who suffer from obstructive sleep apnea based on the PSG result and their lowest score of oxygen saturation level; PSG, polysomnography

Subgroups 1 = PCI, percutaneous coronary intervention; 3 = No intervention
